# Sociotechnical attributes of safe and unsafe work systems

**DOI:** 10.1080/00140139.2015.1009175

**Published:** 2015-04-24

**Authors:** Brian M. Kleiner, Lawrence J. Hettinger, David M. DeJoy, Yuang-Hsiang Huang, Peter E.D. Love

**Affiliations:** ^a^ Department of Industrial and Systems Engineering, Virginia Polytechnic Institute and State University, Perry Street, Blacksburg, VA24061, USA; ^b^ Center for Behavioral Sciences, Liberty Mutual Research Institute for Safety, 71 Frankland Road, Hopkinton, MA01748, USA; ^c^ Department of Health Promotion and Behavior, University of Georgia, 315 Ramsey Center, Athens, GA30602, USA; ^d^ Department of Civil Engineering, Curtin University, GPO Box U1987, Perth, WA6845, Australia

**Keywords:** sociotechnical systems, occupational safety, human–systems integration, macroergonomics, safety climate

## Abstract

Theoretical and practical approaches to safety based on sociotechnical systems principles place heavy emphasis on the intersections between social–organisational and technical–work process factors. Within this perspective, work system design emphasises factors such as the joint optimisation of social and technical processes, a focus on reliable human–system performance and safety metrics as design and analysis criteria, the maintenance of a realistic and consistent set of safety objectives and policies, and regular access to the expertise and input of workers. We discuss three current approaches to the analysis and design of complex sociotechnical systems: human–systems integration, macroergonomics and safety climate. Each approach emphasises key sociotechnical systems themes, and each prescribes a more holistic perspective on work systems than do traditional theories and methods. We contrast these perspectives with historical precedents such as system safety and traditional human factors and ergonomics, and describe potential future directions for their application in research and practice.

**Practitioner Summary:** The identification of factors that can reliably distinguish between safe and unsafe work systems is an important concern for ergonomists and other safety professionals. This paper presents a variety of sociotechnical systems perspectives on intersections between social–organisational and technology–work process factors as they impact work system analysis, design and operation.

## 1. Introduction

For as long as there have been accidents, disasters and near-misses there have also been attempts to explain why and how such events occur. Naturally, the primary goal of these efforts has been to develop the means to help prevent the recurrence of similar incidents. Lessons learned from post-accident investigations have promoted the development of a wide array of analytic tools and approaches to aid in the design, deployment and operation of safe work systems. Furthermore, situations in which an apparently imminent accident was successfully averted, or in which potentially or inherently risky systems maintained consistently safe operations over extended periods of time, have not received the level of attention seen in the literature on accidents. However, the literature on high reliability organisations affords some examples of such an attempt (e.g., La Porte 1996; Weick and Sutcliffe 2007). To what extent are both types of phenomena dependent on similar sociotechnical system properties? Do reliable sociotechnically based discriminators between safe and unsafe work systems exist, and if so what are they and how can they be usefully deployed?

At the outset it is important to note that in using the terms *safe system* and *unsafe system* we do not mean to infer the existence of inherently or irreversibly safe or unsafe work-based sociotechnical systems. Rather, we propose that such systems can be conceived as lying along a dynamic continuum ranging from relatively safe to relatively unsafe depending on a large set of continually evolving, sociotechnical factors that we discuss in this paper. Similarly, it is clearly the case that such systems, having achieved a state of comparative safety relative to some prior state or when compared to other systems, are unlikely to remain safe ‘once and for all’ unless the resources required to maintain and enhance continued safety are invested.

Attempts to identify and understand the underlying characteristics of safe versus unsafe systems vary in many important respects, including (a) the assumed nature and locus of factors considered to underlie either safe work outcomes or accidents (e.g., human behaviour, system component reliability, system design, and organisational policies and procedures) and (b) the level of complexity attributed to the network of events and influences presumed to underlie the occurrence of safe or unsafe system behaviours and outcomes (e.g., Perrow 1984; Dekker 2015; Hollnagel 2014). Among contemporary theorists and practitioners, this diversity of perspectives is manifest in a broad array of theoretical and practice-oriented approaches, several of which are discussed later.

Against this contextual background, we discuss several perspectives on the sociotechnical attributes of safe and unsafe systems, with particular emphasis on factors that impact workplace safety. Sociotechnical attributes are defined as those elements of a work system that correspond to its organisational and technical affordances and constraints, with particular emphasis paid to the complex, dynamic interactions within and between them (e.g., Carayon et al. 2015; Haavik 2011). Simply put, sociotechnical attributes correspond to the social, organisational, technical and work process features of a system considered both as *main effects* and as components of dynamic *interactions*. From a sociotechnical perspective, a system's performance, including its safety performance, emerges from the pattern of dynamic activities within and between its social and technical components.

In recent years, a number of research and practice-oriented approaches towards systems design and safety have adopted an explicitly sociotechnical perspective. These include macroergonomics (Hendrick and Kleiner 2002), cognitive systems engineering (Hollnagel and Woods 1983; Rasmussen, Pejtersen, and Goodstein 1994), human–systems integration (HSI) (Booher 2003; Pew and Mavor 2007), resilience engineering (Hollnagel, Woods, and Leveson 2006), system theoretic accident and modelling processes (Leveson 2012) and sociotechnical systems theory (Coakes and Coakes 2009). While ‘human–systems integration’ is the term most commonly applied to this domain in the USA, ‘human-factors integration’ is the preferred designation within much of the European Union (e.g., Tainsh 2004). Each of these theoretical approaches seeks to shift the focus of research and practice from a traditionally reductionist or ‘microergonomic’ (Hendrick and Kleiner 2002) perspective towards a broader focus on the more systemic level of social, ecological, organisational and technical factors that create and sustain functional (or dysfunctional) work systems and environments. For a review of many these areas within the context of the productivity, health and safety of knowledge workers, we refer the reader to Dainoff (2009).

Formal accident analyses have traditionally focused on engineering design, structural and functional factors, (e.g., component unreliability, software malfunction), apparent human behavioural failures and limitations (e.g., ‘operator error’), or both. In general, the desired outcome is the identification of one or several key points of failure – a definitive ‘root cause’ that can be persuasively argued as having directly precipitated the accident under investigation. The identification of the presumed root cause provides a degree of administrative closure and often leads to efforts geared towards its amelioration through operator training, system redesign, modifications to operational procedures, development of new regulations or any of a host of approaches.

Increasingly, however, incidents involving highly interconnected sociotechnical systems are becoming less amenable to root cause types of explanations (e.g., Leveson 2011; 2012; Goh et al. 2012; Perrow 1984). A well-known example of their limitations occurred in the wake of the *Challenger* disaster when a focus on compromised O-rings (i.e., a failed, technical component) as the proximate cause of failure threatened to mask the overarching influence of powerful cost and timeline pressures on organisational scheduling and launch decision-making (e.g., Vaughn 1997).

In attempting to address the limitations of root cause analysis and similar approaches, a number of researchers and practitioners have begun to examine organisational factors impacting workplace safety (e.g., DeJoy et al. 2010; Huang, Chen, and Grosch 2010; Zohar 1980, 2010, 2011). This work, stemming primarily from the industrial and organisational psychology literature, is based on the fundamental insight that underlying many, if not all, engineering and human behavioural apparent root causes there exists a frequently complex network of organisational and social influences that significantly contributes to creating the conditions under which such failures occur. The current literature on safety climate, discussed in more detail later, is largely a reflection of this emergent area.

HSI, with its roots in systems engineering, and macroergonomics, representing a confluence of traditional human factors and ergonomics (HFE) and sociotechnical systems thinking, also attempts to go beyond root cause, single-point-of-failure approaches to focus on systemic factors whose influences can contribute to the creation of safety risks. These are also more thoroughly discussed later.

### 1.1 The need for a new perspective on workplace safety

The need for an increased understanding of the factors that underlie and promote safety within sociotechnical systems has significantly grown as a direct result of the accelerating complexity of contemporary work environments. As Leveson (2012) has observed, most approaches to systems design and accident analysis that were generally adequate for the simpler work systems of the past are becoming increasingly less useful in light of the significant changes that have occurred in the way such systems are designed and used. Among these are increased organisational and technical system complexity and interconnectedness, the accelerating rate of sociotechnical change in work environments, and the changing nature of workplace risks and accidents.

#### 1.1.1 Increased system complexity and interconnectedness

In contemporary work environments, workers often interact with systems comprising hundreds, or even many thousands of interconnected software, hardware and inter-personal components and sub-systems. In many of these cases, it is effectively impossible for individuals to perceive, let alone comprehend, the activity of each potentially relevant component with respect to its own unique properties and activities, as well as its numerous interactions with other components and sub-systems. The increasingly dynamic nature of contemporary organisational and management systems, as manifest in their tendencies towards globalisation and short- and long-term reorganisation and redistribution of function, further complicates the problem.

#### 1.1.2 The rapid pace of technological change

In the decades following the onset of the Industrial Revolution and throughout much of the post-Second World War era, the broad, sociotechnical characteristics of work generally took many years to substantially change. Work processes and techniques, as well as the social and organisational aspects of the workplace, evolved in an incremental and frequently deliberate fashion. With the onset of the Information Age and its implications for automation and global communications, command and control, the increased prevalence of disruptive technologies (Christensen 1997), and concurrent, dramatic shifts in social and cultural norms (Schwartz 1999), the nature of work began to change in far more rapid, profound and frequently unexpected ways.

As anticipated by the Human Factors Society's Futures Committee in 1980, increasing technological complexity has significantly changed the nature of work in recent decades (Hendrick and Kleiner 2001). Mainframe and personal computing, fibre optic and satellite communications, autonomous and robotic systems, the Internet and many other disruptive innovations have not only changed the technical nature of work, they have also greatly altered its social and organisational nature.

Today society is witnessing transformations in the sociotechnical nature of work unlike anything previously experienced. Groups physically located thousands of miles apart now commonly work together in real time, operators control safety-critical processes and systems by means of computer-mediated interfaces rather than through direct observation and mechanical manipulation, and autonomous and semi-autonomous technologies are an increasingly ubiquitous feature of work-based systems. Clearly, conceptual and analytic approaches intended to maximise the safety of such complex and dynamic systems need to keep pace with the changing nature of work and the factors that constrain and enable it.

#### 1.1.3 The changing nature of accidents

The continuous increase in the complexity of contemporary work systems has fundamentally changed the types of accidents that can and do occur in the workplace. In turn, this has impacted the level of analytic sophistication required to understand the potential for such events, the steps required to prevent them, and the tools and techniques needed to investigate and draw useful lessons from their occurrence.

In the pre- and immediate post-Second World War era, accidents involving mechanical systems were often correctly attributable to simple component failures. In these instances, interactions between mechanical components were generally linear and deterministic as well as readily perceivable and understood. Accidents typically involved the failure of clearly identifiable physical components (e.g., restraining bolts on a boiler that prove to be of insufficient strength to withstand high pressures, resulting in an explosion). In an age in which the number and type of interactions between mechanical and human/organisational components within a work system were comparatively small and whose activities were relatively easy to comprehend, the search for root cause failures was often a fruitful approach. Indeed, even failures in human behaviour could be profitably considered if taken in relation to the adequacy of the physical and organisational systems in place to support adequate performance.

Contemporary work systems have changed greatly. The sheer number and complexity of interactions between hardware, software and human components has exponentially increased the level of difficulty associated with identifying meaningful component failures in an accident (Leveson 2012). A side effect of this growth in complexity has been a corresponding increase in the number and type of engineering and organisational factors underlying human error. As a result of the emergence of these characteristics of complex sociotechnical systems over the past several decades, it has become increasingly clear that traditional approaches to achieving safety within organisations and, by extension, achieving safe systems requires a new way of looking at the problem.

What distinguishes safe from unsafe work systems has not generally changed over time. Safe systems have fewer accidents, and those that do occur are characterised by fewer and less severe injuries to humans and less devastating destruction of physical, information-based and environmental assets than unsafe systems. The problem of identifying the underlying determinants of safe work-based sociotechnical systems is a critical research issue, and one with clear relevance for impacting real-world safety concerns.

## 2. Factors underlying safe and unsafe systems: A historical perspective

Historically, there have been many schools of thought regarding factors that distinguish safe from unsafe work systems. We have chosen to summarise two of the more recognisable and commonly applied approaches: traditional HFE and systems safety. We briefly discuss the assumptions underlying each area's approach to workplace safety, including its benefits, limitations and unique historical contributions. Our intent is to contrast these approaches with macroergonomics, HSI and the concept of safety climate which, as exemplars of the sociotechnical systems approach, we argue are based on more robust assumptions about the factors impacting contemporary workplace safety.

### 2.1 Traditional HFE

Since its inception, HFE has focused primarily on the design of safe and effective human–machine and human–computer interfaces and work processes. HFE has approached these issues from the dual foundations of scientific research on factors that promote or inhibit effective human–system performance, as well as the lessons learned from the application of HFE principles to the design and use of real-world systems (e.g., Stanton et al. 2005; Wickens et al. 2003).

Although systems-based approaches to HFE have been advocated for several decades, culminating recently with respected commentators arguing the case further that systems thinking is vital to HFE's future (e.g. Wilson 2014; Dul et al. 2012), the impact as far as improving workplace safety is concerned has been limited. In addressing workplace safety issues, the tendency for HFE has been to operate at a more molecular (or *microergonomic*) level of individuals or teams interacting with specific tools and technical systems in relative isolation from related organisational and technical influences.

This is not to say that HFE has not had a significantly positive impact on the safe and effective performance of work-related systems and activities. Indeed, prior to the advent of HFE the conception of the human role in accidents and system failures was generally limited to simplistic assumptions concerning human error and failures in training, staffing and/or personnel selection. The modern aircraft cockpit, the iconography and layout of human–computer interfaces and the design of contemporary process control facilities are all examples of the important work that has resulted from the efforts of HFE researchers and designers. Similarly, the useful concepts of physical and mental workload, situation awareness, vigilance decrements and many other vital insights have originated from HFE and continue to exert a powerful influence on safe system design.

In recent years, HFE researchers have begun to focus not only on physical, environmental and behavioural issues in workplace safety, but also on organisational and psychosocial aspects. In addition to factors such as leadership style (Barling, Loughlin, and Kelloway 2002) and supervisor support (Hofmann and Morgeson 1999), well-being has also been shown to predict workplace safety outcomes. For example, Cullen and Hammer (2007) found that strong work performance norms and high work overload were associated with higher work–family conflict. Furthermore, those with lower family-to-work conflict demonstrated increased compliance with safety rules and more willingness to participate in discretionary safety meetings. Similarly, Gyekye (2005) showed that job satisfaction is positively related to safety climate perceptions. Moreover, those with higher job satisfaction were found to be more committed to safety management policies, and consequently were involved in fewer accidents than those with lower job satisfaction.

Similarly, many HFE practitioners have begun to acknowledge that issues related to human–systems design and performance are significantly impacted by the broader sociotechnical context within which they appear (e.g., Clegg 2000; Haslam et al. 2005).

Many contemporary HFE practitioners pursue their research and design efforts on specific system components or sub-systems, or on specific aspects of human–system performance, in relative isolation from the constraints imposed by broader aspects of the sociotechnical system(s) in which their system or phenomena of interest operate. With respect to occupational safety, the result can be meticulously designed, ‘human-centred’ system components (e.g., human–computer interfaces, manual handling operations) that nevertheless perform inadequately within the context of the social and organisational systems within which they are put to use.

### 2.2 System safety

System safety is a discipline that emerged within the engineering and safety communities during the 1940s. Its aim is to design and build safer equipment, frequently by applying lessons learned from accident investigations (O'Keefe 2002). Early work in the area culminated in the publication, in the USA, of Military Standard 882 (MIL-STD-882), a document that has had significant impact on the manner in which safety has been approached in the design, test, production and deployment of large-scale military systems. Currently in its fifth iteration (MIL-STD-882E), the standards set forth in this document continue to exert significant influence on the design of complex systems in the military domain and also in private industry for the development of commercial products such as aircraft, rail transportation, nuclear power and automobiles (Ericson 2005).

One of the major advances of the system safety approach was to formalise the practice of safety engineering within complex system design settings. Design specifically related to ensuring safe system performance, systematic analysis and tracking of potential hazards and specific, safety-oriented system testing are among the practices that have become part of the standard systems engineering repertoire as a result of the introduction of system safety principles (Vincent 2006).

While the discipline of system safety has clearly led to the design, production and deployment of safer complex systems, its focus has primarily been on the prevention of accidents related to *engineering* system failure. To date, it has not significantly expanded its focus to incorporate safety issues related to human interactions with technical systems, nor has it been directly concerned with assessing the impact of organisational characteristics and functions on the safe use of engineering systems. However, Vincent's (2006) system safety text, one of the most widely used in the field, now includes material on ergonomics, representing a potentially important shift in the field towards consideration of human–system performance and safety issues. System safety continues to exert a significant influence on complex systems design, and it can justifiably claim credit for introducing a disciplined approach to safety to the systems engineering process. However, its current lack of emphasis on the human, social and organisational aspects of system safety is a limitation that sociotechnical theories seek to address.

For purposes of this paper, the major point to be made with regard to system safety is that organisations that practice it are, in our view, engaged in a necessary but potentially insufficient approach to enhancing the safety of the systems they design, produce, deploy and/or use. To an extent, adherence to an official military standard, particularly in system design can promote the impression that all that can be done to assure system safety is in fact being addressed. Nevertheless, engineering considerations are only a part, albeit a vitally important part, of the entire spectrum of factors influencing sociotechnical system safety.

## 3. Sociotechnical perspectives on safe work systems

At present there is no single, unified sociotechnical approach to the design and analysis of safe work systems. Instead, as described earlier, there are a number of sociotechnical perspectives that share many common themes. In this section, we describe three contemporary approaches to system design and assessment that are either inspired by or consistent with sociotechnical principles. These are HSI, macroergonomics and safety climate: According to Waterson et al. (2015), HSI is a framework, macroergonomics, and in particular macroergonomic analysis and design (MEAD), is a method, and safety climate is an outcome. We therefore present each in this hierarchical order and describe connections among the three.

- *HSI*: Originally developed as a human-centred, systems engineering discipline, HSI emphasises the centrality of human–system performance and safety criteria for work system design, procurement, deployment and use (Booher 2003). To date, HSI has primarily been employed as a systems engineering framework for the design and deployment of complex military systems, but is beginning to attract interest from workplace safety researchers and practitioners for broader application within industry (e.g., Burgess-Limerick et al. 2011).
- *Macroergonomics*: Most commonly associated with the work of Hendrick and his colleagues, macroergonomics emphasises the critical impact of social and organisational factors on the design of safe and effective work systems, processes and equipment. Through its explicit emphasis on the analysis, design and evaluation of sociotechnical systems, macroergonomics has had an important impact on traditional HFE by emphasising the critical nature of organisational factors in the design of productive and safe work systems and processes (e.g., Hendrick and Kleiner 2002).
- *Safety climate*: A construct originally developed by Zohar and his colleagues (e.g., Zohar 1980, 2010, 2011), safety climate has emerged as a potentially powerful leading indicator of workplace safety (Christian et al. 2009). Having largely emerged from the industrial–organisational tradition within psychology, safety climate is primarily a means of assessing the systemic contributions of organisational processes and communications as they impact safe work performance.

In what follows, we provide an overview of each of these three contemporary approaches, devoting particular attention to their relationships, advantages and shortcomings in accounting for the full spectrum of sociotechnical factors impacting safe work performance. We also discuss the current state of research and practice within each area, and suggest future theoretical and practice-oriented directions.

### 3.1 Human–systems integration

Three Mile Island, Bhopal, Chernobyl and the Challenger disaster are events that represent failures of organisations and technology at the interfaces of people, equipment and work-related processes. The need to address the interfaces between technology and humans was recognised by the US Army during the 1980s. Out of this need emerged HSI, primarily as a systems-engineering approach to the design and deployment of complex systems and only secondarily as a research approach or theory (Booher 2003; Pew and Mavor 2007). HSI has enjoyed varying levels of success, having its major influence primarily in the design of military systems (e.g., Tate et al. 2005).

HSI incorporates a set of processes wherein the tasks, operations and technology in the systems under development are matched to the capabilities and needs of the personnel to be employed within them, while striving to maximise the contribution the entire human–system ensemble makes to operational effectiveness (Strain and Preece 1999). Primarily, HSI assumes that humans are critical elements within a system and that adopting a human-centric perspective towards system design and use increases productivity and safety while decreasing associated costs (Tvaryanas 2006). Its focus has primarily been on enhancing the performance and safety of complex systems by means of the application of nine fundamental design tenets and practices (Booher 2003; US Air Force 2008a):

- *Human factors engineering*: The comprehensive integration of human capabilities and limitations (cognitive, physical, sensory and team dynamic) into system design, to improve human interfaces and to facilitate human performance in training, operation, maintenance, support and sustainment of a system (US Air Force 2008b)
- *Personnel*: The human aptitudes, skills and knowledge, experience levels, and abilities required to operate, maintain and support a system.
- *Training*: The instructional resources required to provide personnel with the requisite knowledge, skills and abilities to properly operate, maintain and support a system.
- *Staffing*: Also commonly referred to as manpower, this area of HSI is concerned with the number and mix of personnel authorised and available to train, operate and support a particular system.
- *Environment*: Includes the conditions in and around the system as well as the concepts of operation constraining its function. Includes requirements necessary to protect the system from the operational environment.
- *Safety*: The application of systems engineering and systems management principles in conducting hazard, safety and risk analysis in system design and development to ensure that all systems, subsystems and their interfaces operate effectively, without sustaining failures or jeopardising the safety and health of operators, maintainers and the system mission.
- *Occupational health*: The consideration of design features that minimise risk of injury, acute and/chronic illness or disability and/or other health-related factors that could reduce job performance of personnel who operate, maintain or support the system.
- *Habitability*: The consideration of factors related to living and working necessary to sustain the morale, health and comfort of the user population. Habitability directly contributes to personnel effectiveness and mission accomplishment, and often includes recruitment and retention issues.
- *Survivability*: The ability of a system, including its operators, maintainers and sustainers to withstand the risk of damage, injury, loss of mission capability or destruction.

Each of these principles and approaches, while typically not explicitly referred to as ‘sociotechnical’ in the HSI literature, is nevertheless highly consistent with such an approach. There are additional guiding principles within HSI, each of which has a strong connection with fundamental sociotechnical concepts. One of these, the importance placed by HSI on emergent human–system performance as a design criterion, is discussed later. Other guiding sociotechnical principles, which HSI shares more closely with macroergonomics and the concept of safety climate, are discussed in the following sections.

#### 3.1.1 Human–system performance as a system effectiveness criterion

Among the most fundamental tenets of HSI is the notion that human–system performance, considered as system-level effectiveness, efficiency and safety, is a central criterion or defining metric in the design or assessment of any complex sociotechnical system. While this might seem tautological, the design of most complex engineering systems with substantial interface to human operators, maintainers and others has generally focused on *technical* system performance as compared to a more operationally relevant focus on overall *human–system* performance. A closely related concept taken from the domain of resilience engineering (e.g., Hollnagel, Woods, and Leveson 2006) concerns the interdependent nature of system effectiveness, efficiency and safety. In this instance, excessive emphasis on one of these performance dimensions at the expense of one or both of the others is likely to result in system performance that fails to successfully achieve any of these performance dimensions over the long run.

HSI's concern with human–system performance impacts system design and assessment in several key respects. For example, complex system design programs generally begin with the formulation of specific design requirements. In system design, if a desired feature or system performance objective is not clearly specified as a design requirement, it is very unlikely that it will be present in the final product unless a painstaking requirement change process is successfully completed. Therefore, a critical aspect of HSI is its focus on the generation of clear human–system performance requirements.

### 3.2 Macroergonomics

Kleiner and Booher (2003) concluded that HSI and macroergonomics share sociotechnical systems theory as a philosophy. HSI, given its roots in systems engineering, is most applicable to the design, development and deployment of major technical systems (e.g. industrial plants), critical technological subsystems (e.g. process control systems) and smaller systems or devices (e.g. human–computer interfaces) (Kleiner and Booher 2003). To date, its focus areas have not included the assessment of organisational influences on system design and use, as in systems ergonomics and macroergonomics. A common application domain for HSI has been the military. Macroergonomics is most applicable to broader sociotechnical systems or ‘very highly complex organizations’, ‘highly complex organizations’ and ‘complex organizations’ (Kleiner and Booher 2003).

In the past, macroergonomics has been viewed as a sub-discipline of HFE that focuses on the analysis and design of work systems (Hendrick and Kleiner 2001). The term ‘work’ is used generically to refer to any form of human effort or activity, but primarily focuses on occupational activity. However, in the area of occupational safety and health, others potentially affected by or who may cause incidents are included in analysis and design. Examples include patients in healthcare systems and drivers who happen upon construction work zones.

The term ‘system’ refers largely to sociotechnical systems and thus, it should be no surprise that the underlying theory behind macroergonomics is sociotechnical systems theory (e.g., Coakes and Coakes 2009). A work system then is one that involves (1) two or more persons, interacting with some form of (2) technology (hardware and/or software, procedures), (3) an internal work environment (both physical and cultural), (4) external environment (with nested sub-environments) and (5) an organisational design and management subsystem. The hardware typically consists of machines, tools and tasks. The internal environment consists of various physical parameters, such as temperature, humidity, illumination, noise, air quality and vibration; it also includes psychosocial and cultural factors. The external environment consists of those elements that permeate the organisation to which the organisation must be responsive to survive and be successful (analogous to environmental forces in biology). Included can be political, regulatory, technological, economic, educational and cultural sub-environments. The organisational design and management of a work system comprises both an organisational structure and the processes by which the work system accomplishes its functions (Hendrick and Kleiner 2001).

Macroergonomics offers a perspective as well as methods and tools for more successful HFE design, development, intervention and implementation (Kleiner 2008) and relates directly to HSI efforts (Kleiner and Booher 2003). Therefore, macroergonomics is an empirical science, a methodology and a perspective. From its foundational research roots in the sociotechnical systems tradition to modern laboratory and field investigations of the relationships among sociotechnical system components, new scientific knowledge about work systems and work system design has emerged, including characteristics of safe systems. We have found that the main gap filled by macroergonomics is that traditional (US) ergonomics does not adequately consider the ‘larger’ system factors and other management systems interventions do not drill down to the interface level.

Systematic macroergonomic methodologies for the analysis, design, and evaluation of work systems such as MEAD, which links sociotechnical analysis (e.g., Emery and Trist 1978) with ergonomics, have also emerged. Finally, macroergonomics represents a perspective that provides the ergonomist with an appreciation for the larger complex system. It is a perspective that increases the likelihood of *traditional* ergonomic interventions having a relatively greater effectiveness than otherwise might be the case (Hendrick and Kleiner 2002). Macroergonomics has been shown to impact a wide range of performance metrics from individual musculoskeletal disorder reduction (Carayon, Smith, and Haims 1999; Robertson et al. 2008) to large-scale organisational culture and performance (Hendrick 1996).

#### 3.2.1 What is the relationship between macroergonomics, HSI and sociotechnical systems?

There are many shared areas of emphasis between macroergonomics and HSI. One of these involves the concept of systems optimisation (versus the maximisation of subsystem components). In macroergonomics, the *joint optimisation* of a sociotechnical system's technical and social/organisational subsystems is pursued. The impact of attending to one subsystem with insufficient regard for the other will often result in system performance problems, as when new technologies and/or work processes are introduced within an organisation without sufficient training, personnel selection and/or user input into their design, selection or implementation (Love and Gunasekaran 1997). In this case, ‘human error’ or other human–system performance problems will frequently arise as a function of the mismatch between technologies, work processes and individual/organisational performance characteristics, capabilities and limitations.

In cases in which new technical systems impact the performance of distributed or co-located work teams or collaborative organisations, the resultant problems can be difficult to predict, often tending to arise under extraordinary and/or stressful situations. The impact of communication failures between New York City police and fire personnel during the terrorist attacks on the USA in 2001 is a particularly tragic example (National Commission on Terrorist Attacks Upon the United States 2004). In this case, incompatible communication systems prevented effective coordination of activities, a state of affairs largely brought about by the historic inability of the two organisations to adopt a common operational and communications framework for large-scale disasters. Certain classes of ‘friendly fire’ fatalities in warfare are a similar example of the consequences of failing to jointly optimise technical and organisational systems (Leveson 2012). In these and similar cases, organisations that make decisions about equipment to be used in the execution of work activities that do not take into account their own organisational and technical constraints and capabilities as well as those of other organisations with whom they must interact are likely to experience significant challenges under conditions of unusual duress.

The importance of adequately accounting for the technical and organisational factors that mutually constrain the nature of work processes is well captured by Ropohl:

The concept of the sociotechnical system was established to stress the reciprocal interrelationship between humans and machines and to foster the program of shaping both the technical and the social conditions of work, in such a way that efficiency and humanity would not contradict each other any longer. (1999, 59)

The principle of joint optimisation then, is operationally defined as the avoidance of maximising any single sociotechnical system component which jeopardises optimisation of the whole system. Maximising the technical subsystem, as exemplified by automating and giving the human leftover functions to perform, may sub-optimise the overall system. Maximising the personnel subsystem, as exemplified by expensive behaviour and attitude modification training without consideration of the organisation's technology or other sociotechnical characteristics, sub-optimises the overall system. Attempting to maximise the organisational design by constantly restructuring without an operationally sound cause (e.g., for purely political reasons) sub-optimises the overall system. Finally, maximising the external environment by allotting too much time with external stakeholders at the expense of internal operations will sub-optimise the total system. Any one of these forms of sub-optimisation can create under-performing work systems. To achieve the appropriate balance then, joint optimisation is operationalised through (a) joint design, (b) a human-centred approach to function and task allocation/design, including consistently accessing the expertise of workers and other key system stakeholders and (c) attending to the organisation's sociotechnical characteristics (Hendrick and Kleiner 2001). In addressing these and similar system design and performance issues, adherence to design principles derived from HSI and macroergonomics can help decrease the likelihood of undesired outcomes including maladaptive human actions (i.e., human error), although these can never be completely eliminated (Hobbs et al. 2008; Love et al. 2011).

User-centred design is important to macroergonomics design and is included in the HSI approach. User-centred design is often discussed in the context of information technology (IT). In the 1980s, there was a growing recognition that traditional methods were inadequate in specifying, designing and implementing IT systems. Researchers like Ken Eason began to investigate ‘multi-user systems’ and ‘co-operative work support systems’ using a socio-technical systems approach (Waterson, Stewart, and Damadaran 2014). However, user-centred design is not limited to IT work systems. Related to ‘participatory design’ (Flach and Dominguez 1995), user-centred design is the notion that representative end-users should be included in all key phases in the design, development, test and deployment of new systems, and that design should support human strengths and limitations.

While user-centred design as applied to the design of technical interfaces is now widely accepted across the HFE community and beyond, its application to the design of organisational systems is largely unique to the field of macroergonomics (e.g., Zink, Steimle, and Schroder 2008) and its European counterpart, systems ergonomics, although it has also been used within HSI approaches to the design of novel ship crew concepts (Tate et al. 2005) as well. Concurrent with the notion of joint optimisation described earlier, participatory design across the entire sociotechnical continuum is an important area of potentially fruitful future improvements in creating and sustaining safe work systems. The prevalence of groups and teams in sociotechnical work systems raises the importance of better understanding the relationship between errors and teams as well (e.g. Rafferty, Stanton, and Walker 2010).

#### 3.2.2 A combined perspective on safer systems

A consistent finding, and one that is fundamental to sociotechnical theory, is that the basic sociotechnical system elements (i.e. subsystems) are mutually interdependent. If a characteristic of one of the five elements discussed in Section 3.2 is changed, it will affect the other four in some way. Thus, if one changes some aspect of the personnel subsystem, it will impact the performance of the technical subsystem, the work system's interaction with the external environment, and the structure and/or processes of the work system. If these influences on the other elements of the sociotechnical system are not anticipated and planned for, the impacts are likely to be dysfunctional and affect the work system in unanticipated and suboptimal ways (Hendrick and Kleiner 2001).

In many ways, macroergonomics is the USA's manifestation of Europe's ‘systems ergonomics’ (Wilson 2014). Nigel Corlett of the UK is attributed with the notion that a larger systems perspective is beneficial in the study and application of ergonomics (Hendrick 1991). Macroergonomics and HSI each adopt a ‘systems ergonomics’. Guided by such a perspective, it is suggested that macroergonomics’ uniqueness centres on its attention to organisational design and management factors, while HSI has enabled the introduction of disciplined, human-centric considerations within the context of traditional systems engineering.

**Figure 1:**
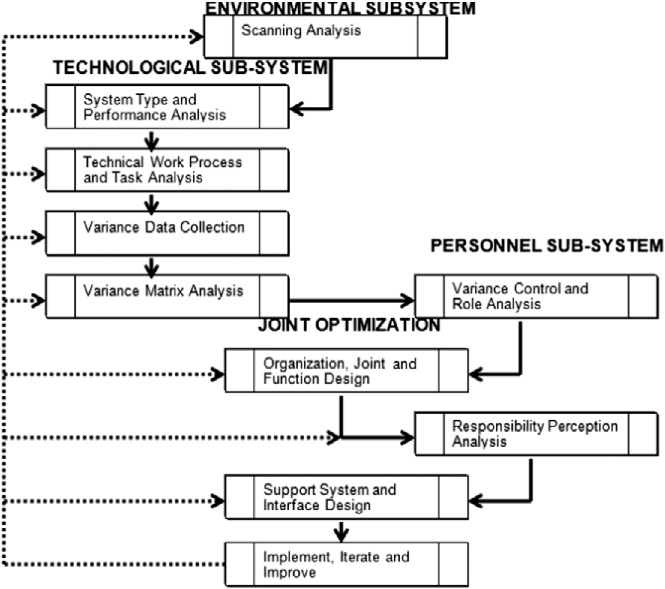
The MEAD process (Kleiner 2006).

Analysis and design efforts of the kind afforded by macroergonomics and HSI are most feasible under four conditions: (1) when developing a new work system, such as when a new organisation is being formed and/or a new complex technology is under development; (2) when a major change to an existing work system is to take place; (3) when a major change occurs in the goals, scope or direction of an organisation or technical system and (4) when the organisation or technical system has a costly chronic challenge that has *not* proven correctable with a purely microergonomic effort, or via other intervention strategies (Kleiner and Booher 2003). In such cases, systems can be improved by means of the MEAD process, as depicted in Figure 1.

As a methodology, it should be noted that MEAD allows the analyst to incorporate tools and techniques from other domains. For example, Jimenez et al. (2012) are integrating simulation and modelling with MEAD to reduce healthcare acquired infections. In this application, simulation is helping to describe and predict the spread of *Clostridium difficile* over time and thus is assisting with environmental subsystem scanning analysis. Patient archival information is collected through electronic patient records and data on healthcare worker activities are obtained through direct observation. Simulation studies will then be designed to test various interventions. Following design of interventions, simulation will also assist in the joint optimisation phase to validate alternatives for organisational, joint and functional design. A similar socio-technical method, SEIPS, has also been applied extensively to healthcare work systems (e.g., Holden et al. 2013). MEAD has also incorporated social network analysis (SNA) in evaluating the personnel system's role network. SNA has been employed by other socio-technical researchers as well (Baber et al. 2013). Also applied to the personnel subsystem evaluation has been cognitive work analysis, another tool utilised by socio-technical researchers (e.g. Read et al. 2014; Stanton 2014).

The ultimate goal of macroergonomics and HSI is a fully *harmonised* work system (at both the macro- and microergonomic levels) which results in improved productivity, job satisfaction, health and safety and employee commitment. Key to the success of this effort is the breaking down of discipline-specific ‘stovepipes’ that frequently exist in system design and development efforts. Simply put, the design of critical system components or sub-systems in relative isolation from one another can result in situations in which joint optimisation becomes difficult, if not impossible to achieve.

### 3.3 Safety climate

Studies of organisations with good versus poor safety performance generally yield sets of attributes and safety program features that include both technical and social/organisational elements (e.g., Cohen 1977; Hale and Hovden 1998; Mearns, Whitaker, and Flin 2003; Saari 1990; Shannon, Mayr, and Haines 1997). Organisations with good safety records not only pay serious attention to risk assessment and hazard control technologies, they also have managers and supervisors who are strongly and visibly committed to safety and employees who are empowered and actively engaged in safety activities. Although this interplay of technical and social/organisational factors is widely acknowledged, broad consensus has not been reached as to the key elements of an effective safety management system (Fernández-Muñiz, Montes-Peon, and Vazquez-Ordas 2007; Robson et al 2007; Hale 2003).

From a sociotechnical systems perspective, and as discussed earlier, a key attribute of safer organisations is the extent to which they jointly optimise the social/organisational and technical aspects of workplace safety. Whether from a social constructionist (Rochlin 1999; Turner and Gray 2009) or systems engineering (Leveson 2012) perspective, safety is an *emergent* *property*, the outcome of interactions among social and technical components of the enterprise in the pursuit of some set of goal-directed activities (mission, core task, etc.). The core feature is joint optimisation – a dynamic state which is constantly subject to change and influence by factors both internal and external to the organisation. External factors are generally beyond an organisation's direct control and include phenomena related to market forces, the regulatory environment, sociopolitical and cultural trends and others. Internal factors are more directly under organisational control and include features such as the number and expertise of workers, pay and other incentive structures, and quantity and quality of available technology with which to conduct work-related activities.

Safety climate may afford a means for assessing the degree of joint optimisation between organisational and technical sub-systems. Safety climate refers to workers’ shared perceptions of their organisation's policies, procedures and practices, as they relate to the value and importance of safety within the organisation (Griffin and Neal 2000; Zohar 1980, 2010, 2011). Safety climate can be seen as an index of an organisation's temporal ‘state of safety’ at a discrete point in time (Cheyne et al. 1998).

Safety climate is sometimes confused with safety culture and, while they are similar concepts, they are usually distinguished in the research literature. Safety culture has been described as shared values and beliefs that interact with an organisation's structures and control systems to produce behavioural norms (Reason, Parker, and Lawton 1998; Thompson et al. 1996; Utall 1983). Safety climate emphasises the workers’ shared perceptions of the organisation's policies, procedures, and practices as they relate to safety within the organisation. In short, safety climate might best be viewed as a measurable surface manifestation of safety culture. The practical and theoretical significance of safety climate as a construct stems from its ability to predict safety behaviour and safety-related outcomes (e.g., accidents and injuries) in a wide variety of settings, as evidenced by diverse samples, in both Western and Eastern cultures (e.g., Cooper and Phillips 2004; Dedobbeleer and Beland 1991; Griffin and Neal 2000; Hofmann and Stetzer 1996; Mearns, Whitaker, and Flin 2003; Niskanen 1994; Oliver et al. 2002; Siu, Phillips, and Leung 2004; Zohar 1980, 2000). The results of several recent meta-analytic studies covering up to 200 published studies (Beus et al. 2010; Christian et al. 2009; Nahrgang, Morgeson, and Hofmann 2011) indicate that safety climate is among the strongest predictors of safety behaviours and injury data in both workgroups and entire companies.

In considering safety climate as a measure of joint optimisation, at least four points merit consideration. First, both the level and strength of safety climate perceptions appear to be relevant. The level of safety climate generally refers to the extent that safety climate perceptions are high or low along some scale or continuum. The strength of safety climate concerns the extent to which the perceptions are shared within and between relevant organisations or work units. The fact that safety climate can be examined at multiple levels adds value to it as a diagnostic tool. Greater agreement or homogeneity across levels indicates a stronger situation or stronger climate. Furthermore, variations across levels might be indicative of disconnects between stated and executed safety policies and procedures. Second, as indicated earlier, safety climate is inherently transient; it reflects the status of an organisation or work system at a particular point in time. While safety climate may not be an end-state, assessment of climate perceptions across time should have predictive significance in terms of showing system improvement, decline or stability.

The third issue involves the extent to which safety climate adequately reflects both sub-systems: the organisational and the technical. If safety climate reflects the overall status of safety within the organisation as perceived by its members, then logically it should be a product of socialtechnical integration, at least to the extent that activities in both sub-systems are visible to employees. Studies have shown that safety-related policies and practices are a strong antecendent of safety climate perceptions (e.g., Isla Dıaz and Dıaz Cabrera 1997; DeJoy et al. 2004, 2010). These policies and practices as enacted create a psychological environment that provides cues about the value and importance of safety and types of behaviours expected. Safety climate may in fact represent the admixture of safety interacting with other organisational tasks and priorities. Management commitment shows up as the single most important component of safety climate perceptions (Flin et al. 2000; Guldenmund 2000; Neal and Griffin 2004), but this does not automatically suggest a reduced role for technical factors. If technical controls are diminished in favour of behavioural or enforcement strategies within an organisation, this should be reflected in judgements of management support and safety climate. The tendency to view safety climate primarily in terms of social and organisational factors may be due to the fact that most safety climate researchers are behavioural scientists. Moreover, the specific interest in safety climate itself arose from the apparent limitations of technically focused solutions to safety problems, and hence the emergence of the need for sociotechnically based approaches.

The fourth point focuses on the diagnostic or prescriptive value of safety climate perceptions or scores. Safety climate scores may indicate the status or trajectory of safety within an organisation, but to what extent do they provide useful information about specific strengths or weaknesses in the overall safety effort? Debate continues as to whether safety climate is a uni-dimensional or multi-dimensional construct (Clarke 2000; Ostroff, Kinicki, and Tamkins 2003; Zohar 2010), and whether any given safety climate measure or questionnaire can be appropriately applied across different industries or business sectors (Zohar 2010). Some have argued that safety climate might best be viewed as an overall or global assessment of safety rather than a diagnostic or audit-type tool (see Murphy, Robertson, and Carayon 2014). A highly positive overall safety climate score may indeed reflect a high degree of social–technical optimisation, but a low score does not reveal the specific nature of any deficiencies or what needs to be done to improve the situation.

In terms of moving ahead, there appear to be three main alternatives. First, safety climate can be retained as a general indicator of safety status within an organisation and as one component of a multi-faceted safety program. Safety climate's value as a leading indicator of safety performance would justify assigning it a sentinel type function, but it would not automatically have any diagnostic or prescriptive value in terms of safety program improvement. The second alternative would be to continue to develop customised multi-dimensional safety climate measures for specific industries that would better capture context specific attributes that could aid problem identification and the targeting of prevention and control strategies. The third alternative is somewhat of a compromise between the first two. This would involve drawing upon applicable theory to improve and broaden the diagnostic value of safety climate. For example, sociotechnical theory has given rise to various analytic strategies such as the MEAD process. As we have noted previously, MEAD represents a combination of sociotechnical systems theory and macroergonomics (Robertson, Kleiner, and O'Neill 2002) that builds upon the idea of joint optimisation, also directly relevant to HSI-based approaches to system design. As a theory-derived method, MEAD provides an analytic process that could be conceptually linked to safety climate as presently discussed.

A second theory-based approach would be to build upon existing dual process or balance models of work design. Models such as Job Demands-Resources Model (Bakker and Demerouti 2007) or the Effort-Reward Imbalance Model (Siegrist 1996) have been applied across a wide variety of work situations and could provide a starting point for devising an analytic framework or for constructing a more generalisable and diagnostic safety climate tool. In terms of conceptual space, the relative balance of job demands and resources may not be that distinct from the idea of joint optimisation. In addition, these two sets of working conditions appear relevant to virtually any occupational context. A recent meta-analysis of over 200 independent samples has demonstrated the relevancy of job demands and resources to safety-related outcomes (Nahrgang, Morgeson, and Hofmann 2011). Job Demands-Resources Model also features two processes, health impairment and motivation, which may represent key mechanisms through which working conditions impact safety outcomes.

In summary, the argument is presented that safety climate may reflect the level of joint optimisation within work organisations, and that joint optimisation is central to effective safety performance and may underlie safety climate's demonstrated utility as a leading indicator of safety performance.

## 4. Conclusion: recommendations for workplace systems design and use

Our intent in this paper was to examine sociotechnical attributes of safe and unsafe systems, where ‘system’ refers to a work-centric ensemble of integrated social–organisational and technical components. To do so, we approached the issue from the three sociotechnical perspectives with which we as individual researchers are most experienced: HSI, macroergonomics and safety climate. While each area has its unique attributes, areas of emphasis and applications, all share a common sociotechnical perspective; specifically, that the emergent characteristics of work systems, of which safety is the principal current focus, are a function of the dynamic characteristics of component properties and interactions. Our intent has been to examine sociotechnical system properties that can help distinguish safe from unsafe systems.

A sociotechnical perspective on workplace safety affords researchers and practitioners a systems-based theoretical and practice-oriented framework within which to envision, study and work to alleviate safety risks and hazards, particularly within complex work settings. Major themes within sociotechnical systems thinking illuminate several key areas for research and practice with the ultimate goal of providing effective guidance to support the design and deployment of safe and effective work systems and to assess existing work systems with an eye towards sustainment or improvement.

We have discussed *joint optimisation* as a key sociotechnical construct of potential significant value to these endeavours. As noted by Pasmore (2001, 41) in a review of early sociotechnical systems thinking, joint optimisation is central to the sociotechnical systems approach: ‘No matter how advanced the technology, it would fail if not mated with a social system designed to operate the technology effectively. This principle, known as joint optimisation, was to become the cornerstone of sociotechnical systems theory’. There is much that is intuitive about the notion of joint optimisation, but also much that needs to be clarified for it to be put into effective practice. For example, what does it mean to operationalise joint optimisation across the life cycle of a work system? For work-based systems in the design and development phase, the goal of joint optimisation is clearly relevant to the development of adequate system requirements. Lessons learned from HSI and macroergonomics have shown that technical and organisational design requirements should be approached in a coordinated fashion for maximum design efficiency and system effectiveness, particularly for systems whose operation is heavily dependent upon tight, efficient couplings between technical and human capabilities and limitations, and for which the potential consequences associated with inadequate performance are high. The manner in which these types of requirement analyses and decisions should be approached is still an unsettled issue, though at its core the process involves an examination of problems associated with proper allocation of function at the ‘sharp end’ of system performance, and the development of an organisational support system focused on safe and effective outcomes (see Challenger, Clegg, and Shepherd 2013). Of course, issues of joint optimisation do not end at the requirements development phase. As individual work systems evolve in response to changing macroeconomic, sociocultural, political and legislative/regulatory conditions there is an ongoing need to assess the degree to which the social–organisational and technical aspects of the system are correctly aligned with respect to new operational ‘realities’. MEAD offers one macroergonomics-based approach to accomplishing this important task. Safety climate assessments, in addition, afford a valuable means of assessing the coherence of organisational policies and practices related to safety which, as the nature of the work system changes, are also likely to evolve. What the safety climate literature demonstrates is the criticality of adequately managing change such that sensible and practical safety policies and practices are kept up to date with evolving operational conditions and, perhaps most critically, consistently communicated and practiced across the organisation.

Fundamentally, sociotechnical approaches to workplace safety emphasise the critical importance of design across the sociotechnical continuum. Simply put, the sociotechnical perspective stresses the importance of accounting for the impact of systems design and function across all sociotechnical levels, from the ‘microergonomic’ level of the individual workstation human–machine interface to the macroergonomic level of organisational system design and function. Given the dramatic growth in complexity of everyday work systems, sociotechnical systems thinking can perhaps most usefully be thought of as providing a set of constructs, objectives and methods to assist vital efforts towards joint optimisation of system components and interrelationships.
